# Gene Expression and Cytokine Profile Correlate With Mycobacterial Growth in a Human BCG Challenge Model

**DOI:** 10.1093/infdis/jiu615

**Published:** 2014-11-07

**Authors:** Magali Matsumiya, Iman Satti, Agnieszka Chomka, Stephanie A. Harris, Lisa Stockdale, Joel Meyer, Helen A. Fletcher, Helen McShane

**Affiliations:** The Jenner Institute, University of Oxford, United Kingdom

**Keywords:** BCG, transcriptomics, cytokines, vaccines, tuberculosis

## Abstract

***Background.*** Bacillus Calmette-Guerin (BCG) vaccine is the most widely administered vaccine in the world, yet its mechanism of action remains unclear. We hypothesize that certain immune pathways are associated with reduced mycobacterial growth following BCG challenge in human volunteers.

***Methods.*** We used samples from a mycobacterial challenge in which previously BCG-vaccinated or BCG-naive adults in the United Kingdom were challenged intradermally with a standard dose of BCG. Any remaining BCG was quantified in a skin biopsy specimen obtained 2 weeks after challenge and used as a measure of BCG growth and functional antimycobacterial immunity. We measured the immune response over the 2-week challenge, using DNA microarrays and flow cytometry, and correlated this with mycobacterial growth.

***Results.*** The magnitude of the immune response to BCG is greater in previously vaccinated volunteers, and this correlates with reduced mycobacterial growth but increased scarring at the vaccination site. In particular, the interferon γ and interleukin 17 pathways are strongly induced in previously vaccinated volunteers and correlate with reduced mycobacterial growth in this population.

***Conclusion.*** This study identifies pathways associated with control of mycobacterial growth in vivo in human volunteers and supports the use of BCG challenge as a tool for evaluating vaccine efficacy and identifying mechanisms of antimycobacterial immunity.

Tuberculosis is a major global health problem, with an estimated 8.6 million cases and 1.3 million deaths in 2012 [1]. Effective vaccination is likely to be necessary for the long-term control of the tuberculosis epidemic. However, bacillus Calmette-Guerin (BCG) vaccine, the only currently licensed vaccine for the prevention of tuberculosis, provides variable protection against pulmonary disease [2], and in tuberculosis-endemic countries, the incidence of tuberculosis remains high despite widespread BCG coverage. Research efforts into new tuberculosis vaccines have focused largely on 2 strategies: (1) modify BCG or replace it with an attenuated strain of *Mycobacterium tuberculosis*, or (2) improve on the protection of BCG through prime-boost regimens, often using viral vectors expressing *M. tuberculosis* antigens, to enhance the memory cells primed by vaccination with BCG [3]. Twelve novel tuberculosis vaccines are currently in clinical trials [1]. The results of the first efficacy trial of a novel vaccine, modified vaccinia virus expressing antigen 85A (MVA85A), were published in early 2013 [4] and showed no enhanced protection, compared with BCG alone, in South African infants. Despite these advances, the development of new vaccines against tuberculosis remains hampered by the difficulty in evaluating efficacy. The frequency of new *M. tuberculosis* infection is very low, even in high-burden settings, making efficacy trials long and expensive. Although animal models exist, none exhibit all stages of human disease, and the extent to which they accurately predict protection in humans is unclear. Because of the tissue damage caused by tuberculosis and the difficulty in ensuring complete clearance of infection, human challenge with *M. tuberculosis* is currently not ethically possible. The clinical trial from which samples in this study were collected was conducted as part of an effort to create a human model of mycobacterial growth and its control, using BCG as a challenge organism. The trial included 4 groups who received the following vaccination regimes before BCG challenge: A, none; B, MVA85A; C, BCG (≥6 months prior to the trial); and D, BCG (≥6 months prior to the trial) followed by MVA85A 4 weeks before challenge. Groups B and D, who received MVA85A, were not included in this study, but the original group names have been retained here. The primary analysis of the trial showed a reduction in BCG growth in the previously BCG-vaccinated groups, compared with the BCG-naive groups, and that BCG growth was inversely correlated to the interferon γ (IFN-γ) enzyme-linked immunosorbent assay (ELISPOT) response to purified protein derivative tuberculin (PPD-T) [5]. In this study, we used flow cytometry and gene expression analysis to identify biological correlates of mycobacterial control in this setting, using stored samples from the trial.

## MATERIALS AND METHODS

### Study Design

Samples used in this study were obtained from a phase 1 trial (clinical trials registration: NCT01194180), which was approved by the Medicines and Healthcare Products Regulatory Agency (EudraCT 2010-018425-19) and the Oxfordshire Research Ethics Committee A (reference 10/H0505/31). The study design was described in detail by Harris et al [5].

Groups included in this study are group A (BCG naive) and group C (BCG vaccinated; median time since vaccination, 10 years). All volunteers were intradermally challenged with a standard vaccine dose of BCG (SSI, Statens Serum Institut); 0.1 mL containing 2 × 10^5^–8 × 10^5^ colony-forming units [CFU]) as previously described [5]. A single operator performed skin biopsies on the BCG challenge site of all volunteers 2 weeks after challenge, as previously described [6]. All biopsy specimens were processed, DNA was extracted, and quantitative polymerase chain reaction (qPCR) was performed as previously detailed [5] and described below. Peripheral blood mononuclear cells (PBMCs) for gene expression analysis were collected and cryopreserved as previously described [7] on the day of challenge (day 0) and days 2, 7, and 14 after challenge. Whole blood specimens for cytokine analysis were collected on days 0, 2, and 14 and processed as described below.

### BCG Quantification by PCR

Biopsy specimens were snap frozen on the day of challenge and later thawed and homogenized in 1 mL of sterile phosphate-buffered saline (PBS). Homogenate was thawed, and BCG DNA from 200 µL of homogenate was released using the tough microorganism lysing kit (Precellys) in a Precellys 24 machine by shaking 3 times at 6500 rpm for 30 seconds each. Homogenate was transferred to a separate tube, and 50 µL of PBS was used to wash the remaining homogenate from the beads. Next, 180 µL of animal tissue lysis buffer and 20 µL of proteinase K (Qiagen) were added, vortexed, and incubated at 56°C for 4 hours. From this point, the extractions were performed as previously described [5, 6]. qPCR primers ET 1 and ET 3 were used for detection of BCG DNA. The sequences used are given in the article by Harris et al [5]. PCR reactions were performed as previously described [5, 6], using BCG-naive macaque tissue homogenate as a negative control. A standard curve was obtained by extracting BCG DNA from 1 in 10 serial dilutions of 5 pooled vaccine vials in PBS and correcting for live BCG from the corresponding CFU on solid agar.

### Gene Expression Analysis

Cryopreserved PBMCs were rapidly thawed in a 37°C water bath and transferred to a 15-mL Falcon tube containing 10 mL of R10 (Roswell Park Memorial Institute medium with 10% fetal calf serum, 1% l-glutamine, 1% Pen-Strep, and 1% sodium pyruvate). PBMCs were pelleted, and supernatants were discarded and resuspended in 10 mL of R10 with 20 µL of Benzonase (Merck Chemicals) and rested overnight at 37°C in 5% CO_2_. PBMCs were counted on a Casy Counter (Roche), and 2 × 10^6^ cells were stimulated for 12 hours with either R10 medium alone or containing 1 × 10^6^ CFU of BCG (Statens Serum Institute). After 12 hours, supernatant was removed, and the PBMCs were resuspended in 350 µL of RLT buffer (Qiagen) containing 10 µL/mL β-mercaptoethanol and frozen at −20C.

RNA was extracted using the RNeasy kit (Qiagen) according to manufacturer's instructions, including the optional protocol for DNA digestion (RNase-free DNase kit, Qiagen). Messenger RNA was amplified from the total RNA, using the Illumina Totalprep kit (Ambion) according to manufacturer's instructions. RNA quantity and quality was assessed using a Nanodrop ND-1000 Spectrophotometer and an Agilent Bioanalyzer (Agilent RNA 6000 Nano Kit). A total of 750 ng of amplified complementary RNA was labeled and hybridized to Illumina Human HT-12 v4 beadchips as specified in the manufacturer's instructions. Beadchips were scanned on an Illumina iScan machine, and data were extracted using the GenomeStudio software.

### Stimulation of Whole Blood

Mycobacteria-specific intracellular cytokines (ICS) were measured in whole-blood samples as previously described [8]. Briefly, blood samples were incubated with 1 µg/mL αCD28 and 1 µg/mL αCD49d (BD) and stimulated with 20 µg/mL PPD (SSI, Denmark) and 5 µg/mL staphylococcal enterotoxin B (Sigma Aldrich); unstimulated blood samples served as negative controls . Stimulated and unstimulated blood samples were incubated at 37°C in 5% CO_2_ for 6 hours, 3 µg/mL Brefeldin A (Sigma Aldrich) was added, and cells were incubated for another 6 hours in a timed water bath. Whole-blood samples were then treated with 2 mM ethylenediaminetetraacetic acid (Gibco), and red blood cells were lysed using FACS Lysing solution (BD). Samples were frozen for batched ICS analysis.

### ICS Analysis

Stimulated and fixed whole-blood samples were permeabilized and incubated with antibodies against CD3 (AF700), tumor necrosis factor α (TNF-α; peridinin chlorophyll protein–cyanine 5.5 [Cy5.5]) and IFN-γ (phycoerythrin [PE]–Cy7) from Ebioscience. CD4 (allophycocyanin [APC]), CD14 (Pacific Blue), and interleukin 17 (IL-17; AF488) from Biolegend. CD8 (APC-H7) was obtained from BD, and interleukin 2 (IL-2; PE) was obtained from Beckman Coulter. Samples were acquired on an LSRII (Becton Dickinson). Responses were analyzed using FlowJo (Tree Star), and cytokines were measured in singlet CD14^−^CD3^+^ cells, CD4^+^ T cells, or CD8^+^ T cells and in singlet CD3^−^CD14^+^ cells. The gating strategy is specified in Supplementary Figure 3. Presented results are percentages of cytokine-expressing cells minus responses in unstimulated cells. Polyfunctional cytokine immune responses were analyzed using Spice software (http://exon.niaid.nih.gov/spice/). The monocyte to lymphocyte ratio was calculated as the ratio of singlet CD14^+^CD3^−^ cells to singlet CD3^+^CD14^−^ cells.

### Microarray Analysis

Raw Illumina probe data were exported from Beadstudio and screened for quality, using the R package arrayQualityMetrics [9]. Gene expression data were analyzed using the bioconductor platform in R [10]. Genes not expressed above background levels in any sample were removed (*P* < .05). In limma [11, 12], background correction and quantile normalization were performed using the neqc function [13]. Probes with an interquartile intensity range of < 0.3 (log_2_ transformed) across all samples were filtered using bioconductor's genefilter package. Lists of differentially expressed genes were generated using limma (*P* value cutoff, .05 after Benjamini-Hochberg correction [11–13]). Pathway analysis was performed using the Internet-based tool DAVID (Database for Annotation, Visualisation and Integrated Discovery) [14]. Heat maps were created in R, using the gplots package [15].

**Figure 1.**
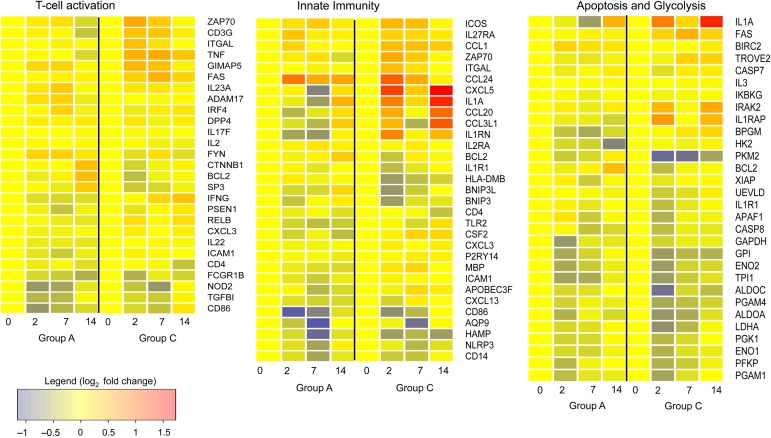
Bacillus Calmette-Guerin (BCG) vaccination induces changes in gene expression in unstimulated peripheral blood mononuclear cells (PBMCs). Heat map showing changes in expression of genes associated with T-cell activation, innate immunity, apoptosis, and glycolysis over the 14-day challenge period in unstimulated PBMCs for the BCG-naive group (*A*) and the previously BCG-vaccinated group (*C*). Median log_2_ fold changes from day 0 are shown.

Genes selected for inclusion in Figure 1 were selected as they contributed most highly to the enrichment of the gene ontology categories shown in Table 1. The genes in Table 2 were selected to include those from each group (A and C) with the highest fold changes (and equivalent values for the other group, if it was only highly differentially expressed in one group). A few additional genes, such as the one encoding TNF, are included for interest because of their importance to the field.

**Table 1.** Gene Ontology Terms Associated With Bacillus Calmette-Guerin (BCG) Vaccination

Study Group, Gene Ontology Term	Genes, No. (%)	*P* Value	
		Unadjusted	Adjusted^a^
Group A			
Immune response	33 (10.9)	4.50E-08	8.10E-05
Regulation of cytokine production	14 (4.6)	6.40E-06	5.70E-03
Defense response	26 (8.6)	1.50E-05	9.30E-03
Chemical homeostasis	23 (7.6)	2.20E-05	1.00E-02
Homeostatic process	27 (8.9)	1.50E-04	5.40E-02
Group C			
Establishment of protein localization	84 (7)	8.70E-07	5.70E-04
Cell activation	42 (3.5)	7.40E-07	6.10E-04
Glycolysis	15 (1.3)	7.00E-07	7.60E-04
Leukocyte activation	38 (3.2)	4.80E-07	1.60E-03
Intracellular transport	72 (6)	5.30E-06	2.50E-03
T-cell activation	23 (1.9)	1.20E-05	4.40E-03
Lymphocyte activation	30 (2.5)	2.10E-05	6.20E-03
Hexose catabolic process	16 (1.3)	2.10E-05	6.70E-03
Glucose metabolic process	25 (2.1)	3.20E-05	7.90E-03
Monosaccharide catabolic process	16 (1.3)	3.00E-05	8.00E-03
Positive regulation of apoptosis	49 (4.1)	8.30E-05	1.90E-02
Immune system development	35 (2.9)	1.40E-04	2.40E-02
Induction of apoptosis	39 (3.3)	1.30E-04	2.50E-02
T-cell differentiation	14 (1.2)	1.80E-04	2.50E-02
Hexose metabolic process	27 (2.3)	1.90E-04	2.50E-02
Generation of precursor metabolites and energy	38 (3.2)	1.70E-04	2.60E-02
Phosphate metabolic process	90 (7.5)	2.20E-04	2.90E-02

Differentially expressed genes over the 2-week challenge period were determined using linear modeling in limma. The lists of differentially expressed genes for each group were analyzed using DAVID to identify significantly enriched gene ontology terms.

^a^ By the Benjamini–Hochberg method.

**Table 2.** Changes in Gene Expression Following Bacillus Calmette-Guerin (BCG) Stimulation Are Higher in Group C, Compared With Group A

Probe Identifier	Symbol	Challenge				Day 14			
		Group A		Group C		Group A		Group C	
		Adjusted *P*	Fold Change	Adjusted *P*	Fold Change	Adjusted *P*	Fold Change	Adjusted *P*	Fold Change
ILMN_1726448	MMP1	9.90E-23	31.86	3.87E-34	145.70	3.36E-19	24.30	4.13E-27	60.08
ILMN_1699651	IL6	5.44E-13	42.12	2.03E-25	138.98	3.10E-13	25.03	2.16E-18	35.90
ILMN_2158713	IL1F9	1.89E-31	99.69	1.57E-47	130.36	7.15E-30	112.53	6.32E-41	63.45
ILMN_1663866	TGFBI	1.05E-31	−71.21	1.32E-43	−128.73	8.92E-31	−89.07	1.84E-41	−120.62
ILMN_2207291	IFNG	3.63E-21	46.31	4.40E-39	127.96	6.78E-24	118.38	6.26E-40	188.75
ILMN_1668063	FCN1	1.55E-26	−74.84	1.30E-46	−127.53	6.71E-25	−81.87	1.10E-41	−81.58
ILMN_1773245	CCL3L1	6.58E-18	39.48	7.79E-33	103.31	9.39E-17	44.33	2.42E-28	28.85
ILMN_1661861	CSF2	2.03E-22	54.93	6.57E-48	100.97	1.20E-21	69.61	1.47E-43	70.41
ILMN_1658483	IL1A	5.26E-11	29.16	2.15E-23	85.06	5.96E-10	16.83	7.75E-17	32.09
ILMN_1657234	CCL20	5.74E-11	18.08	2.87E-24	83.86	1.04E-10	18.74	1.48E-16	31.41
ILMN_2203271	FPR3	2.85E-38	−48.24	1.55E-52	−83.48	9.00E-36	−47.24	9.44E-52	−92.77
ILMN_1774685	IL24	1.82E-11	21.50	9.31E-20	48.26	1.74E-15	10.32	9.82E-16	9.80
ILMN_1686623	CSF1R	4.44E-23	−15.60	1.25E-34	−46.77	1.49E-24	−24.18	9.56E-32	−39.03
ILMN_1797009	F3	2.07E-13	5.70	8.03E-32	36.83	1.35E-14	7.72	3.69E-26	13.33
ILMN_1815205	LYZ	3.87E-22	−20.81	1.84E-29	−34.57	1.46E-20	−21.98	1.19E-24	−22.82
ILMN_1703538	AIF1	2.49E-19	−9.61	1.54E-44	−29.73	4.01E-18	−10.35	1.20E-36	−14.82
ILMN_1671509	CCL3	1.95E-17	22.48	5.86E-32	29.60	6.49E-16	23.22	4.06E-25	16.24
ILMN_1653766	CCL24	2.87E-14	−14.44	3.26E-26	−28.07	2.61E-15	−22.36	4.22E-26	−33.30
ILMN_1735910	VMO1	9.90E-23	−16.89	9.60E-31	−25.46	2.19E-19	−13.95	1.56E-27	−20.95
ILMN_1730816	GPR162	3.87E-22	−14.88	2.09E-40	−22.33	2.52E-21	−17.42	1.96E-38	−21.49
ILMN_1780533	RNASE6	1.76E-19	−11.65	6.18E-32	−22.15	1.93E-18	−12.90	8.09E-29	−18.72
ILMN_1728106	TNF	4.94E-19	8.29	9.26E-37	18.69	3.36E-19	10.40	3.89E-33	15.27

Gene expression was determined for each group, and at each time point, between BCG-stimulated and unstimulated peripheral blood mononuclear cells. Changes in gene expression were determined using linear modeling in limma, including the volunteer as a factor.

### Statistical Analysis

Statistical analysis was performed using Prism (GraphPad) software. The Mann–Whitney *U* test was used to compare cytokine responses between the 2 study groups. Differences were considered statistically significant at *P* values of < .05.

### Determination of the BCG Scar Severity Rank

Photographs of the BCG injection challenge site were taken on day 14, immediately before biopsy, to document each participant's reaction to BCG. A high degree of heterogeneity in local reactions to BCG was noted. The photographs were ranked continuously, from least to most severe, by the trial physician subsequently through the end of the trial but before the quantification of BCG in biopsy specimens. The ranking took account of size, redness, and swelling of the inflammatory reaction at the site of injection and is specified in Supplementary Figure 1.

## RESULTS

### Immune Response to BCG Is Weaker in BCG-Naive Volunteers

BCG induces changes in gene expression of circulating PBMCs over the 2-week period following BCG challenge. Twenty-four volunteers, 13 with a prior history of BCG vaccination, were challenged with BCG, and blood specimens were collected immediately before challenge (day 0) and 2, 7, and 14 days later. We determined gene expression in unstimulated PBMCs and identified genes within the groups at each time point that were differentially expressed relative to baseline. Both groups showed changes in expression of genes related to the immune system during the challenge, but the fold change and the number of significantly differentially expressed genes were higher in the previously vaccinated group (group C). A total of 1500 genes were differentially expressed, compared with baseline, over the challenge period in group C, whereas 500 were differentially expressed in group A. In both groups, differentially expressed genes showed enrichment of genes associated with the immune response, T-cell activation, glycolysis, and apoptosis, but these changes were stronger in group C than in group A (Table 1 and Figure 1). The greatest number of differentially expressed genes was seen 2 days after challenge, although for genes involved in T-cell activation the peak occurred later.

For all volunteers, we also determined gene expression profiles of PBMCs stimulated for 12 hours with BCG. As in the unstimulated samples, differentially expressed genes in both groups reflected a strong innate component in the immune response to BCG. However, fold changes were much higher in group C, showing a role for memory responses in increasing the magnitude of the immune response to BCG. Fold changes for BCG-stimulated PBMCs compared with unstimulated PBMCs, are shown for a subset of genes on days 0 and 14 (the times of BCG challenge and biopsy, respectively) for the 2 groups in Table 2.

**Figure 2.**
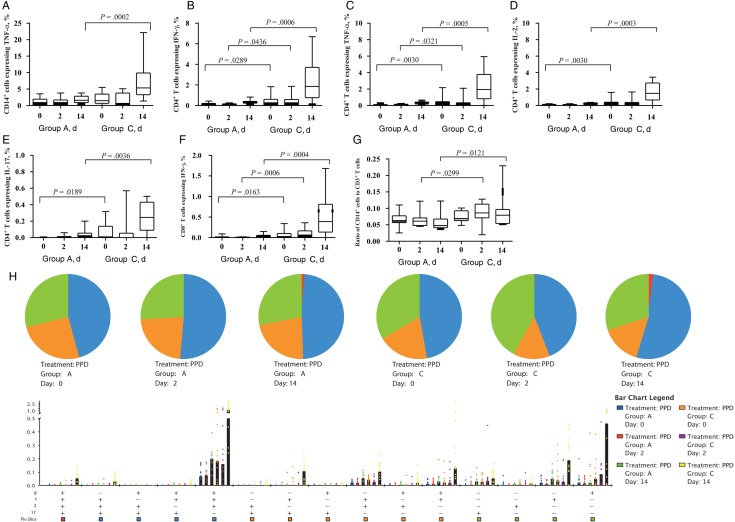
Mycobacteria-specific whole-blood cytokine responses and the ratio of CD14^+^ cells to CD3^+^ T cells in volunteers in study groups A and C on the day of bacillus Calmette-Guerin (BCG) challenge (day 0) and days 2 and 14 after challenge. *A*–*F*, Percentages of CD14^+^ cells, CD4^+^ T cells, or CD8^+^ T cells making cytokines. *G*, Ratio of CD14^+^ cells to CD3^+^ T cells in volunteers in the 2 study groups. *H*, Polyfunctional CD4^+^ T-cell cytokine responses. The pie charts illustrates proportions of CD4^+^ T cells making 1, 2, 3, or 4 cytokines, whereas median percentages of CD4^+^ T cells making these cytokine are shown in the bar chart. *P* values for differences in frequencies of polyfunctional CD4^+^ T cells between groups A and C at day 14 are shown in the table. Box and whisker plots show median values, interquartile ranges, and minimum and maximum values. Abbrevations: IFN-γ, interferon γ; IL-2, interleukin 2; IL-17, interleukin 17; PPD, purified protein derivative; TNF-α, tumor necrosis factor α.

We determined PPD-specific cytokine responses in whole-blood samples collected from volunteers in groups A and C at days 0, 2, and 14 after BCG challenge (Figure 2*A* and 2*G*). Frequencies of CD14^+^ cells producing TNF-α were comparable at baseline, indicating preexisting innate immune response to BCG in the 2 study groups. At day 14, there were more CD14^+^ TNF-α–expressing cells in group C, compared with group A. Study of the lymphocyte cytokine profile in the 2 groups revealed that levels of CD4^+^ T cells producing IFN-γ and TNF-α and CD8^+^ T cells producing IFN-γ were significantly higher in group C, compared with group A, at all 3 time points. Additionally, levels of CD4^+^ T cells producing IL-2 and IL-17 were significantly higher in group C volunteers at days 0 and 14.

As monocytes are considered an important population of cells in which mycobacteria reside, while lymphocytes are known to be the major effector cells in tuberculosis immunity, we investigated the effect of BCG challenge on the ratio of monocytes to lymphocytes (defined as the ratio of CD14^+^ cells to CD3^+^ T cells in the unstimulated samples). This ratio was significantly higher in group C at days 2 and 14 after challenge than in group A (Figure 2*H*). Finally, polyfunctional CD4^+^ T cells were detected in both groups, with group C showing significantly higher frequencies of CD4^+^ T cells making multiple cytokines simultaneously (Figure 2*I*).

**Figure 3.**
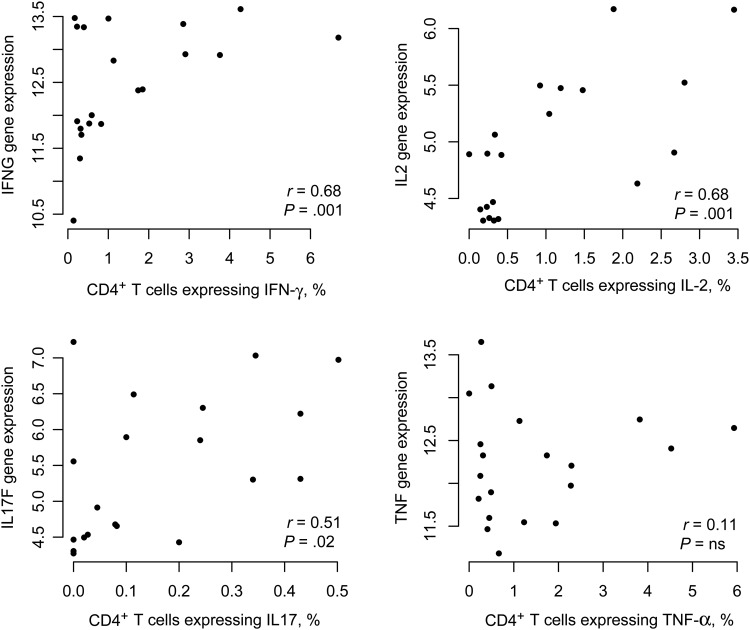
Correlation of cytokine expression, measured by gene expression microarray and intracellular cytokine staining. Cytokine production following bacillus Calmette-Guerin (BCG) stimulation (measured by DNA microarray) or purified protein derivative stimulation (measured by flow cytometry) were determined. Pearson correlation analysis was performed between values obtained by DNA microarray and the percentage of cytokine-positive CD4^+^ T cells. Abbreviations: IFN-γ, interferon γ; IL-2, interleukin 2; IL-17, interleukin 17; NS, not significant; TNF-α, tumor necrosis factor α.

We next correlated cytokine production quantified by flow cytometry of PPD-stimulated whole blood with expression of the same cytokines measured by microarray analysis of BCG-stimulated PBMCs (Figure 3). The percentage of CD4^+^ T cells expressing IL-2, IFN-γ, and IL-17 correlated with the microarray values obtained for these genes (IFN-γ: Pearson *r* = 0.68, *P* = .001; IL-2: Pearson *r* = 0.68, *P* = .01; and IL-17: Pearson *r* = 0.51, *P* = .02). By contrast, the values for TNF-α production did not correlate with either TNF-α–expressing CD4^+^ T cells or TNF-α–expressing CD14^+^ cells (data not shown), perhaps reflecting production of TNF by a greater variety of cell types.

### BCG Growth Correlates Inversely With Scar Severity and Cytokine Production

**Figure 4.**
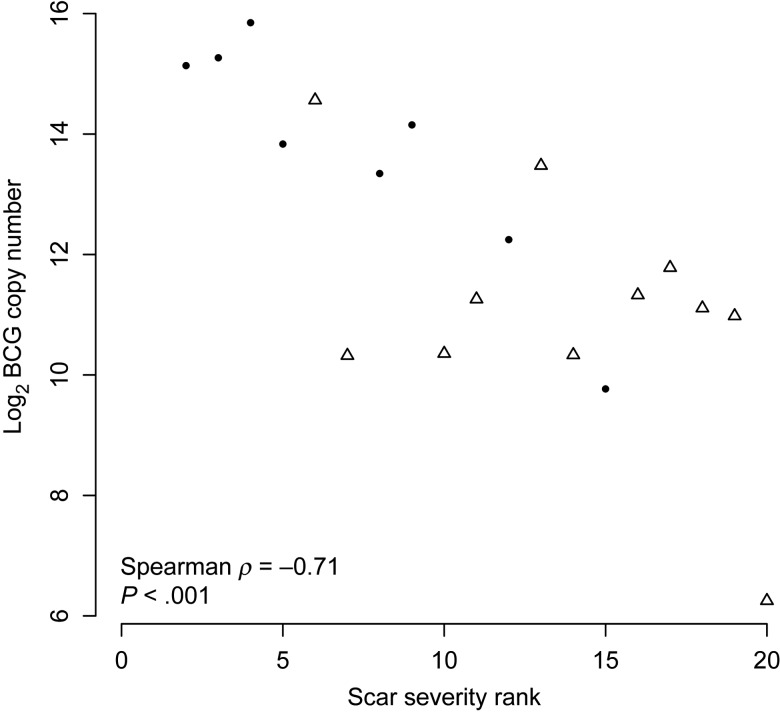
Scar severity correlates inversely with mycobacterial growth. Mycobacterial growth was measured by quantitative polymerase chain reaction analysis of bacillus Calmette-Guerin (BCG) in biopsy samples obtained from the site of BCG injection 14 days after vaccination. Scar severity was determined by ranking photos of the vaccination site taken immediately before biopsy. The ranking of the severity of local reactions was performed by the trial clinician before quantification of BCG in the biopsy specimens. Group A, open triangles; group C, filled circles. Spearman *ρ* = −0.71, *P* < .001.

BCG growth was measured by qPCR of the biopsy specimen, and these data have been previously reported [5]. Photos of the BCG vaccination site were taken immediately before biopsy, and these were ranked to give a measure of severity of the local reaction (termed “scar severity”). The photos were ranked by the trial clinician before determination of the BCG burden in the biopsy specimen by qPCR. Ranked photos are shown in Supplementary Figure 1. There was an inverse correlation observed between scar severity and BCG growth (Spearman *ρ* = −0.71, *P* < .001; Figure 4).

Expression of cytokines and related genes, as measured by both flow cytometry and DNA microarray, were correlated to BCG growth and scar severity values (Table 3). Figures showing the correlations of cytokines and selected genes with BCG growth and scar severity are also shown in Supplementary Figure 2. The inflammatory cytokines IFN-γ, IL-17, IL-2, interleukin 22 (IL-22), interleukin 23 (IL-23), and CXCL3 showed an inverse correlation with BCG growth and a positive correlation with scar severity. By contrast, the pattern-recognition receptor NOD2 and members of the leukocyte immunoglobulin-like receptor (LILR) family correlated in the opposite direction. Additionally, in polyfunctional T cells, the production of the following combinations of cytokines at day 14 showed a correlation with inhibition of BCG growth: IFN-γ, TNF-α, and IL-2 (*P* = .0007); TNF-α, IL-2, and IL-17 (*P* = .0127); IFN-γ and TNF-α (*P* = .0125); and TNF-α and IL-17 (*P* = .0091). The response to BCG/PPD stimulation changed over the course of the challenge, with the fewest correlations seen 2 days after BCG, reflecting the evolving immune response.

**Table 3.** Correlation Between Cytokine Production and Bacillus Calmette-Guerin (BCG) Growth or Scar Severity

Variable	Day 0 (Challenge), *P* (*r*)^a^		Day 2, *P* (*r*)^a^		Day 7, *P* (*r*)^a^		Day 14 (Biopsy), *P* (*r*)^a^	
	BCG Growth	Scar	BCG Growth	Scar	BCG Growth	Scar	BCG Growth	Scar
Flow cytometry								
CD4^+^ IFN-γ^+^	NS	NS	NS	.0102 (0.5737)	ND	ND	<.0001 (−0.7754)	.002 (0.6614)
CD4^+^ TNF-α^+^	NS	NS	NS	.0139 (0.5538)	ND	ND	<.0001 (−0.7675)	.0017 (0.6702)
CD4^+^ IL-2^+^	NS	NS	NS	.0073 (0.5940)	ND	ND	<.0001 (−0.7860)	.001 (0.6924)
CD4^+^ IL-17^+^	NS	NS	NS	NS	ND	ND	.0063 (−0.552)	NS
CD8^+^ IFN-γ^+^	NS	NS	NS	NS	ND	ND	<.0001 (−0.7233)	.0013 (0.6819)
Gene expression								
CXCL3	.003 (−0.6)	.018 (0.5)	NS	NS	NS	NS	NS	NS
NOD2	.001 (0.6)	NS	NS	NS	NS	NS	.024 (0.5)	NS
FCGR1B	.03 (0.5)	NS	NS	NS	NS	NS	.01 (0.6)	NS
IL10RB	.0003 (0.7)	.035 (−0.5)	NS	NS	NS	.045 (−0.4)	NS	NS
IL17F	.011 (−0.5)	.006 (0.6)	NS	NS	.002 (−0.7)	.017 (0.6)	<.0001 (−0.8)	.006 (0.7)
F3	.04 (−0.4)	NS	.015 (−0.5)	NS	NS	NS	.031 (−0.5)	NS
FAS	.018 (−0.5)	.018 (0.5)	NS	NS	.026 (−0.5)	.013 (0.6)	NS	.044 (0.5)
LILR A5/A6/B2	.01 (0.5)	.02 (−0.5)	NS	NS	.01 (0.5)	.02 (−0.5)	.01 (0.6)	.02 (−0.5)
IL5	NS	.029 (0.5)	NS	NS	.014 (−0.5)	.024 (0.5)	.005 (−0.6)	.003 (0.7)
IFNG	NS	.02 (0.5)	NS	NS	.003 (−0.6)	.014 (0.6)	.031 (−0.5)	NS
IL2	NS	NS	.011 (−0.6)	.016 (0.6)	.024 (−0.5)	.027 (0.5)	.003 (−0.6)	NS

Cytokine production was measured in peripheral blood mononuclear cells stimulated by purified protein derivative/BCG, with the background value (unstimulated) subtracted. BCG growth and scar severity were determined 14 days after challenge.

Abbreviations: BCG, Bacillus Calmette-Guerin; IFN-γ, interferon γ; IL-2, interleukin 2; IL-17, interleukin 17; LILR, lymphocyte immunoglobulin-like receptor; ND, assay not done; NS, not significant; TNF-α, tumor necrosis factor α.

^a^ *P* values are not corrected, and all correlations are Spearman *r*. Correlations were performed for all 24 volunteers, separately for each day.

## DISCUSSION

BCG has been administered to >3 billion people since its introduction >90 years ago. During this time, the prevalence of tuberculosis has fallen dramatically in some countries, but in others it is higher than ever. The use of a human BCG challenge model as a surrogate of protection against which to test novel vaccines may prove a valuable tool in early selection of promising vaccine candidates [5, 6]. BCG is known to be highly effective in the United Kingdom, giving around 80% protection [16]. The aims of the study were to characterize the kinetics of the immune response to BCG in naive and previously vaccinated volunteers and to look for correlations between these immune parameters and the number of mycobacteria recovered from the site of injection at the end of the challenge period.

The immune response in unstimulated and stimulated cells in both groups showed activation of innate immunity to BCG. In the previously vaccinated volunteers, however (group C), the fold changes were much higher, indicating that prior exposure to BCG increases the magnitude of the immune response. These observations are consistent with previous microarray studies done in naive and BCG-vaccinated mice challenged with BCG [17]. Although the 2 groups showed a degree of overlap in differentially expressed genes, fold changes were much higher in the vaccinated mice. Additionally, similar pathways were modulated in both human volunteers and mice. BCG challenge in previously BCG-vaccinated humans caused the monocyte to lymphocyte ratio to increase, compared with that in BCG-naive subjects, which is likely to affect the gene expression measured by microarray. The significance of this change is not clear and could be caused, for example, by proliferation of monocytes following BCG vaccination.

The BCG challenge model allows associations to be made between different immune parameters and BCG growth. In this trial, previously BCG-vaccinated volunteers had significantly lower amounts of BCG recovered from the challenge site, compared with BCG-naive volunteers, consistent with the protective effect of BCG in this population. This was therefore a good opportunity to identify potential correlates of mycobacterial control. Genes in which a change in expression in the stimulated as compared to unstimulated samples was associated with BCG growth included *IFNG* and *IL17F*, together with other genes associated with these 2 cytokines, such as *NOD2, IL22, IL23A*, and *FCGR1B*. Several recent studies have reported important roles for these cytokines in protection from *M. tuberculosis* infection. IFN-γ is known to be necessary, although not sufficient, for protection, and recent studies also suggest an important role for T-helper type 17 (Th17) cells and the IL-23/IL-17 pathway. The latter can provide partial protection from *M. tuberculosis* challenge and have been shown to be necessary drivers of Th1 immunity and IFN-γ responses in the face of interleukin 10 production during infection [18–20]. In cattle, IL-22 and IFN-γ production by PPD-stimulated PBMCs were identified as the primary predictors of vaccine induced protection in a *Mycobacterium bovis* challenge model [21]. Additionally, a recent article identified NOD2 as a crucial component of epigenetic reprogramming of monocytes following BCG vaccination, which led to nonspecific protective effects [22]. In this study, levels of polyfunctional CD4^+^ T cells were significantly higher in BCG-vaccinated volunteers 2 weeks after BCG challenge, and cells producing multiple cytokines have previously been shown to be associated with protection in a *Leishmania major* model [23]. Here, we show a negative correlation between BCG growth and frequencies of CD4^+^ T cells producing combined cytokines (IFN-γ, TNF-α, and IL-2; TNF-α, IL-2, and IL-17; IFN-γ and TNF-α; and TNF-α and IL-17) 14 days after challenge.

In this study, the identification of T cells producing IL-2 and IL-17 in previously BCG-vaccinated volunteers before BCG challenge may suggest the presence of a pool of central memory T cells, a response that is then boosted following BCG challenge. Other studies have previously shown that BCG can induce central memory T-cell responses in different populations [24, 25]. Unfortunately, markers to identify memory populations of T cells were not included in these experiments, and exploring this would require further testing. It is unlikely that time since BCG vaccination can explain the data, because we have not seen a correlation between response to BCG by IFN-γ ELISPOT and time since BCG vaccination in this or any other previous studies. Finally, although our data show an inverse correlation between cytokine production and BCG growth, in a previously published efficacy trial in South African infants, increased cytokine production in response to PPD stimulation in BCG- and MVA85A-vaccinated infants was not associated with protection [4]. However, this may be because the magnitude of the response was much lower in South African infants. Furthermore, differences in age and population make it difficult to draw parallels between the 2 studies.

The increase in local adverse events following a second BCG vaccination is well documented and was also seen in this study [6, 26, 27]. The severity of local inflammation correlated with the immune response (*IL17F, IFNG, FCGR1B*) and increased control of mycobacterial growth. The balance between bacterial killing and excessive inflammation is at the core of the relationship between humans and *M. tuberculosis.* These data show that too little inflammation fails to control mycobacterial growth, whereas a protective immune response comes at the cost of collateral damage. BCG has been shown to be effective in protecting the United Kingdom population from tuberculosis, suggesting that the spectrum seen here in group C may indicate an optimal balance. In Malawi, where BCG is not protective, vaccinated infants develop smaller scars, weaker Th1 responses, and stronger Th2 and regulatory responses in response to PPD-T stimulation, compared with their United Kingdom counterparts [28]. Likewise, laboratory mice in Brazil and Mexico show less susceptibility to infection with *M. tuberculosis* and also less protection from BCG [29]. Although the data shown here and recent studies suggest a role for the IL-17 pathway in the development of a protective response, excessive activation of this pathway has also been associated with detrimental tissue damage in this and other diseases [18, 30, 31], and the level of this cytokine's importance in vaccine-induced immunity is presently unknown. The human BCG challenge model may become a powerful tool in future vaccine research, both in terms of vaccine evaluation and for the identification of potential immune correlates of protection. An important next step will be to test this model and characterize the response to BCG in populations where BCG is not effective, which may yield insight as to why these differences exist and how they relate to control of mycobacterial growth.

## Supplementary Data

Supplementary materials are available at *The Journal of Infectious Diseases* online (http://jid.oxfordjournals.org). Supplementary materials consist of data provided by the author that are published to benefit the reader. The posted materials are not copyedited. The contents of all supplementary data are the sole responsibility of the authors. Questions or messages regarding errors should be addressed to the author.

Supplementary Data
