# Supplementary material for: Gene Expression and Cytokine Profile Correlate With Mycobacterial Growth in a Human BCG Challenge Model
Source: J Infect Dis. 2014 Nov 7;211(9):1499–509. doi: 10.1093/infdis/jiu615 (PMC4392868; doi:10.1093/infdis/jiu615)
Supplement: Supplementary Data [file supp_211_9_1499__index.html]

Gene Expression and Cytokine Profile Correlate With Mycobacterial Growth in a Human BCG Challenge Model — Gene Expression and Cytokine Profile Correlate With Mycobacterial Growth in a Human BCG Challenge Model — Supplementary Data 

# Gene Expression and Cytokine Profile Correlate With Mycobacterial Growth in a Human BCG Challenge Model

## Supplementary Data

Supplementary Data

**Files in this Data Supplement:**

- Supplementary Figure 1 - pdf file
- Supplementary Figure 2 - pdf file
- Supplementary Figure 3 - pdf file
- Supplementary Data - Docx file
