# Supplementary figures and images for: Gene Expression and Cytokine Profile Correlate With Mycobacterial Growth in a Human BCG Challenge Model
Source: J Infect Dis. 2014 Nov 7;211(9):1499–509. doi: 10.1093/infdis/jiu615 (PMC4392868; doi:10.1093/infdis/jiu615)

## Supplementary figure S1

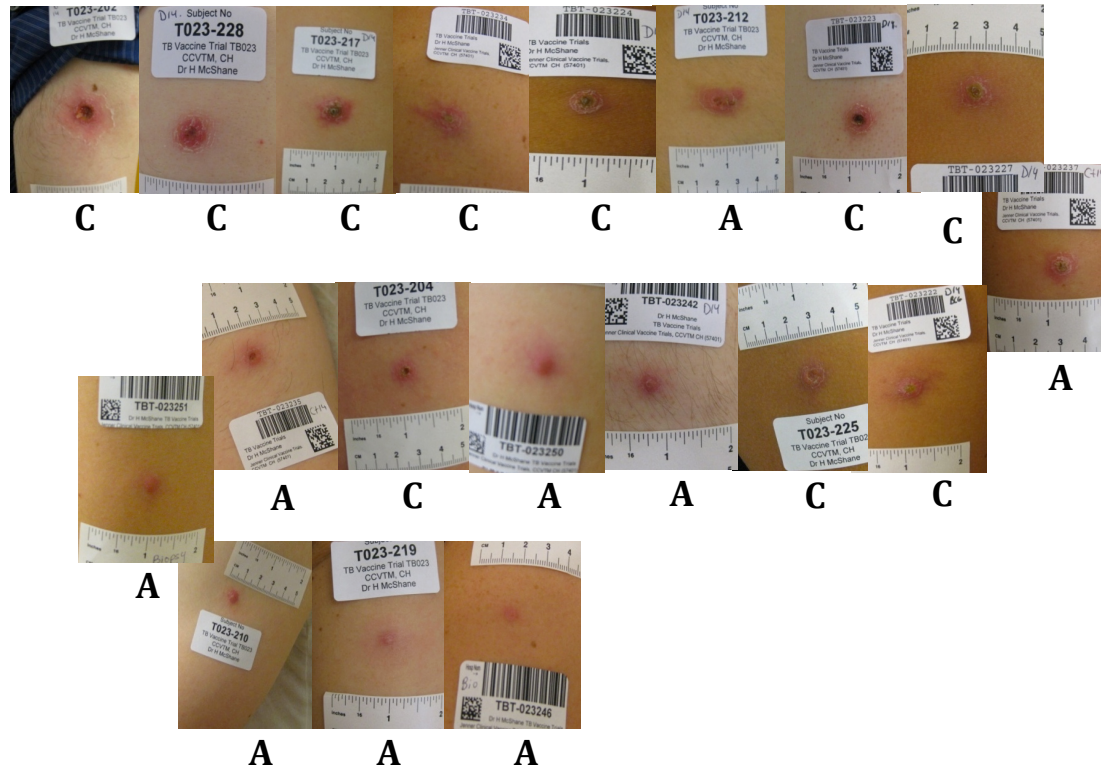

Supplement: Supplementary Data [file supp_jiu615_jiu615supp_fig1.pdf]

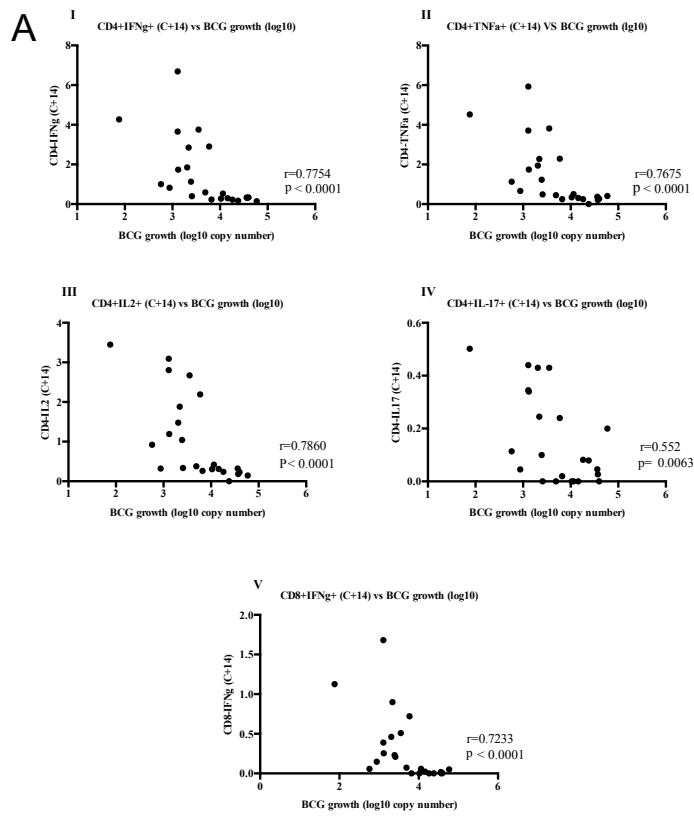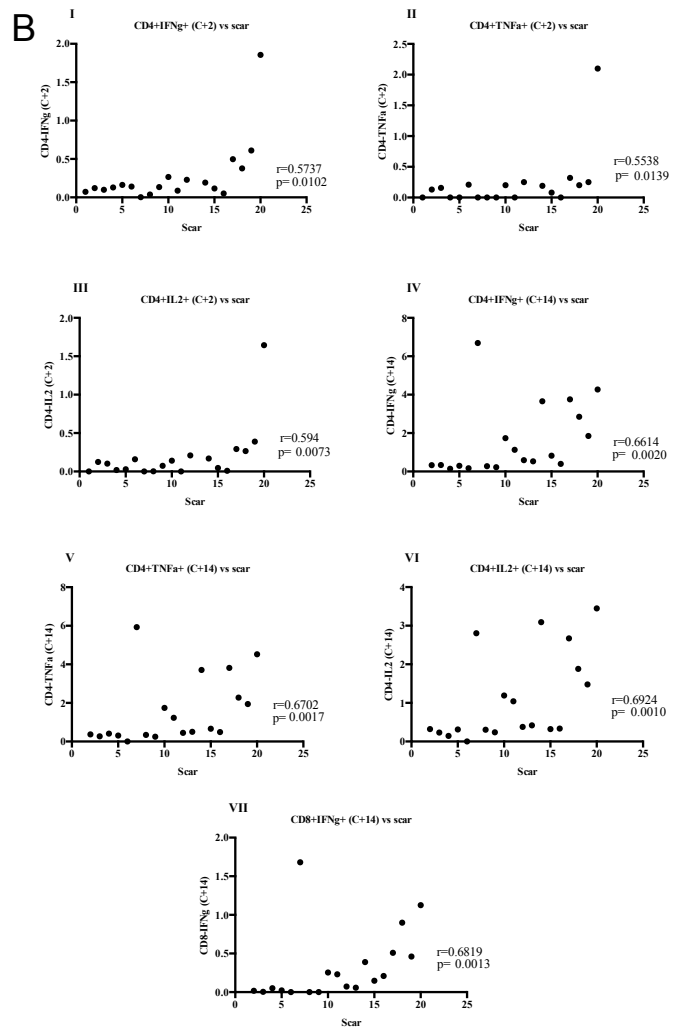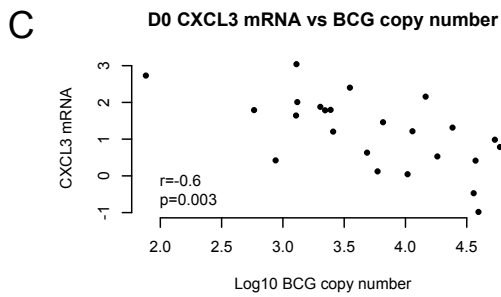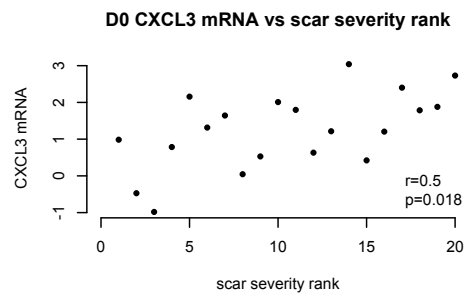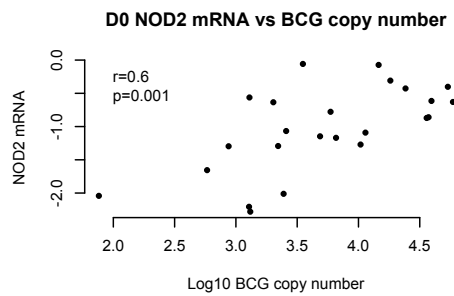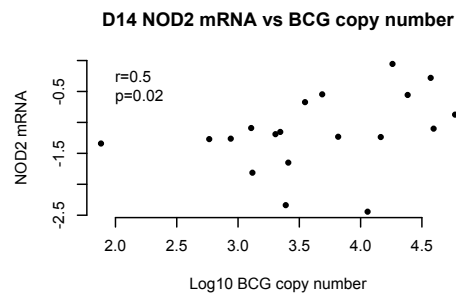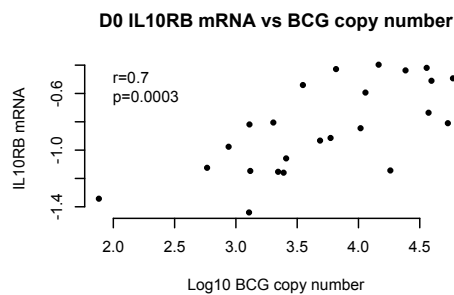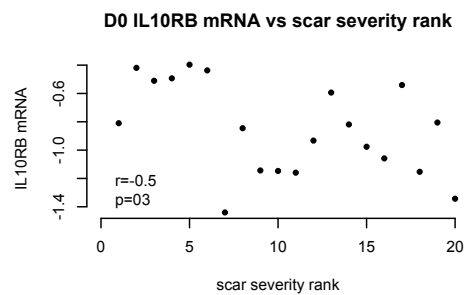

Supplement: Supplementary Data [file supp_jiu615_jiu615supp_fig2.pdf]

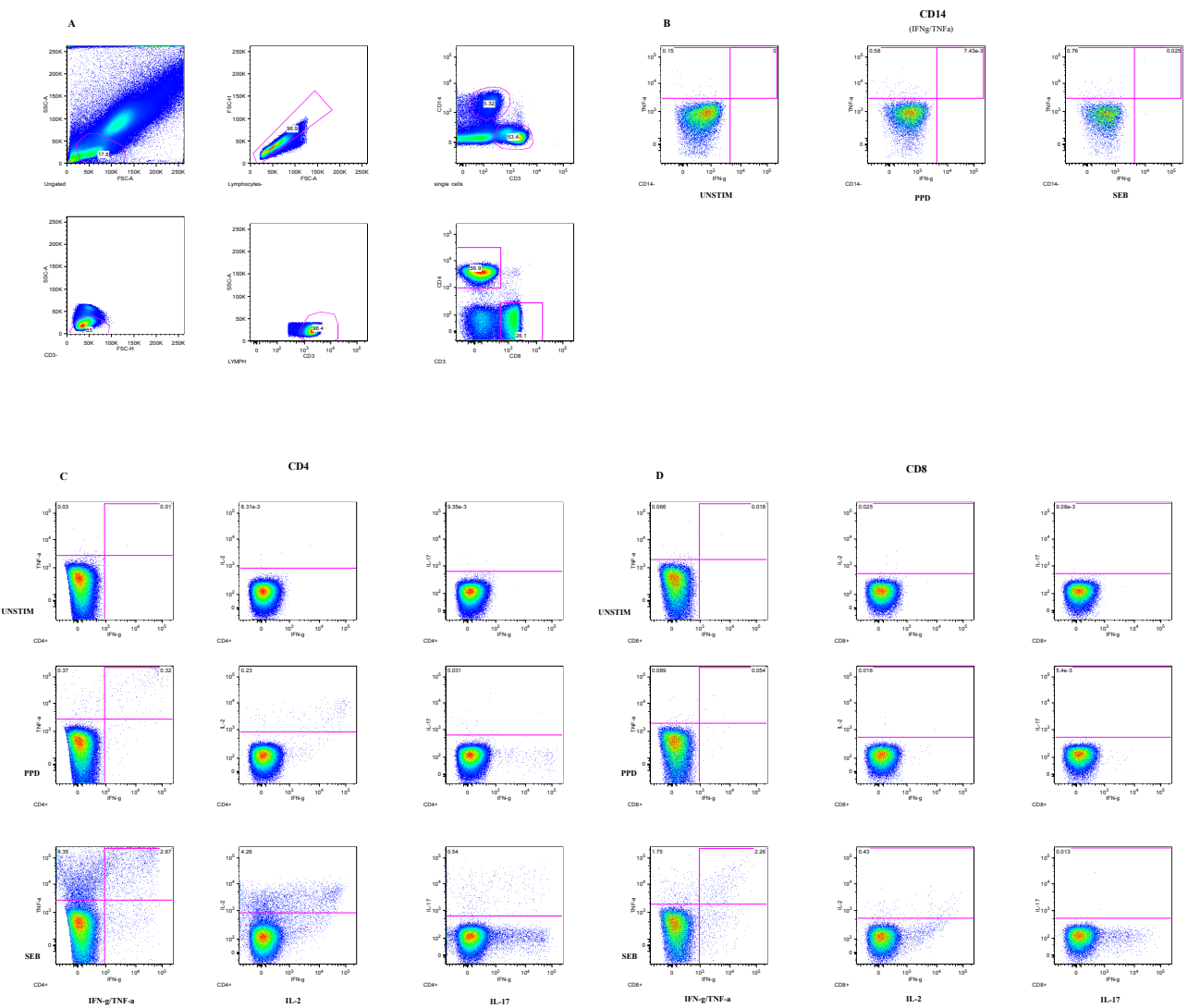

Supplement: Supplementary Data [file supp_jiu615_jiu615supp_fig3.pdf]
