# Supplementary material for: Gene Expression and Cytokine Profile Correlate With Mycobacterial Growth in a Human BCG Challenge Model
Source: J Infect Dis. 2014 Nov 7;211(9):1499–509. doi: 10.1093/infdis/jiu615 (PMC4392868; doi:10.1093/infdis/jiu615)
Supplement: Supplementary Data [file supp_jiu615_jiu615supp.docx]

Supplementary Figure S1: BCG scar severity rank. Photos of the BCG vaccination site were taken immediately prior to biopsy on day 14 from the majority of volunteers. These were ranked according to severity of local reaction by the trial clinician (most severe, top left). Letters indicate the vaccination group of the volunteer.

Supplementary Figure S2: Correlations between cytokine production and scar severity or BCG growth. Panel A (I to VII) show correlation between scar severity and whole blood ICS cytokines. Panel B (I to V) represent correlations between BCG growth copy number and whole blood ICS cytokines. P and r values are show on each plot. Panel C represents correlations between gene expression and scar severity or BCG growth. Legends show r and p values for Spearman’s rank correlation test.

Supplementary Figure S3: Gating strategy for flow cytometry analysis

Panel **A:** lymphocytes were gated on side scatter versus forward scatter plots, gating on singlet cells followed this, then CD14+ and CD3+ cells were gated. Gating on CD14+ cells cytokines is shown in panel B. T lymphocytes were further characterized by gating on small lymphocytes followed by CD3 expressing cells, and then CD4+ and CD8+ T cells. Panel **B s**hows CD14+ cytokines on unstimulated and PPD- and SEB-stimulated cells. Panels **C** and **D** show cytokines detected on stimulated and unstimulated CD4+ and CD8+ T cells respectively.
